# Modulation of Keap1/Nrf2/ARE Signaling Pathway by Curcuma- and Garlic-Derived Hybrids

**DOI:** 10.3389/fphar.2019.01597

**Published:** 2020-01-28

**Authors:** Melania Maria Serafini, Michele Catanzaro, Francesca Fagiani, Elena Simoni, Roberta Caporaso, Marco Dacrema, Irene Romanoni, Stefano Govoni, Marco Racchi, Maria Daglia, Michela Rosini, Cristina Lanni

**Affiliations:** ^1^ Department of Drug Sciences, University of Pavia, Pavia, Italy; ^2^ Scuola Universitaria Superiore IUSS, Pavia, Italy; ^3^ Department of Pharmacy and Biotechnology, University of Bologna, Bologna, Italy; ^4^ Department of Pharmacy, University of Napoli Federico II, Naples, Italy

**Keywords:** curcumin, Nrf2, Keap1, NQO1, HO-1, dimethyl fumarate, miRNAs

## Abstract

Nrf2 is a basic leucine zipper transcription factor that binds to the promoter region of the antioxidant response element (ARE), inducing the coordinated up-regulation of antioxidant and detoxification genes. We recently synthesized a set of new molecules by combining the functional moieties of curcumin and diallyl sulfide, both known to induce the expression of antioxidant phase II enzymes by activating Nrf2 pathway. The aim of the study is to investigate the ability of such compounds to activate Keap1/Nrf2/ARE cytoprotective pathway, in comparison with two reference Nrf2-activators: curcumin and dimethyl fumarate, a drug approved for the treatment of relapsing-remitting multiple sclerosis. Furthermore, since Nrf2 pathway is known to be regulated also by epigenetic modifications, including key modifications in microRNA (miRNA) expression, the effects of the hybrids on the expression levels of selected miRNAs, associated with Nrf2 signaling pathway have also been investigated. The results show that compounds exert antioxidant effect by activating Nrf2 signaling pathway and inducing the ARE-regulated expression of its downstream target genes, such as HO-1 and NQO1, with two hybrids to a higher extent than curcumin. In addition, some molecules induce changes in the expression levels of miR-125b-5p, even if to a lesser extent than curcumin. However, no changes have been observed in the expression levels of mRNA coding for glutathione synthetase, suggesting that the modulation of this mRNA is not strictly under the control of miR-125b-5p, which could be influenced by other miRNAs.

## Introduction

Nrf2 (NF-E2-related factor 2), a member of the Cap’n’collar (CNC) transcription factor family, is a redox-sensitive transcription factor that plays a key role in adaptation to cellular stress. Under normal homeostatic conditions, Keap1 anchors the Nrf2 transcription factor within the cytoplasm targeting it for ubiquitination and degradation by 26S proteasomes (Niture et al., 2014). Under stress conditions, phosphorylation and/or redox modification of critical cysteines residues in Keap1 inhibits the enzymatic activity of the Keap1-Cul3-Rbx1 E3 ubiquitin ligase complex (Tebay et al., 2015). Consequently, free Nrf2 translocates to the nucleus, where it dimerizes with Maf proteins (musculoaponeurotic fibrosarcoma) and binds to the antioxidant response element (ARE), also called electrophile response element (EpRE), a *cis*-acting enhancer sequence located in the promoter region of a battery of downstream genes encoding cyto-protective, antioxidant, and phase II detoxifying enzymes or proteins, such as NAD(P)H: quinone reductase-1 (NQO1), heme oxygenase-1 (HO-1), and glutathione synthetase (GSS) (Tebay et al., 2015). The Nrf2/Keap1/ARE signaling pathway can be activated by various exogenous and endogenous small molecules (Baird and Dinkova-Kostova, 2011; Paunkov et al., 2019) and controls also the expression of genes involved in the regulation of cell proliferation and survival (Malhotra et al., 2010).

Natural products have emerged as a great source of bioactive compounds with health beneficial impact. One example are polyphenols, phenolic compounds that act on biological systems exerting protective effects not only by direct antioxidant capacity, but also by interacting with signal‐transduction pathways that regulate transcription factors and, consequently, the expression of genes and proteins (Auclair et al., 2009; Spencer, 2010; Camargo et al., 2010). Among the variety of pathways, it has been demonstrated that polyphenols such as curcumin, hydroxytyrosol contained in olive oil, resveratrol, and epigallocatechin-3-gallate extracted from green tea could modulate the transcription factor Nrf2, *via* translocation into the cell nucleus and induction of the expression of its target genes (Scapagnini et al., 2011; Xicota et al., 2017; Martínez-Huélamo et al., 2017)

In our previous papers, we described and characterized the ability of a set of new curcuma- and garlic-derived compounds to inhibit Aβ oligomerization and fibrilization (Simoni et al., 2016; Simoni et al., 2017). The main structure of these hybrids combines the diallyl sulfide (DAS), which represents the mercaptan moiety of garlic-derived organosulfur compounds, and the hydroxycinnamoyl group, a recurring chemical function of polyphenols, such as curcumin, rosmarinic acid, and coumarin (Ho et al., 2012; Witaicenis et al., 2014; Nabavi et al., 2015a). Our data demonstrated the ability of these molecules to act as scavenger agents in presence of oxidant stressors (Simoni et al., 2016; Simoni et al., 2017). In particular, we identified a catechol derivative (compound 1, see **Table 1**), with remarkable anti-aggregating ability and antioxidant properties (Simoni et al., 2016). Starting from the results obtained with compound 1, which is considered the lead compound, its structure was systematically modified by focusing on the aryl substitution pattern, the thioester function, and the aliphatic skeleton with the aim of strategically tuning the pharmacological profile (Simoni et al., 2017). Herein, to investigate the structure-dependent activation of intracellular defensive pathways, we focused on a selection of these hybrids (compounds 1–6, **Table 1**). Two reference molecules, known to activate Nrf2 pathway, were used for comparison: curcumin (CURC) and dimethyl fumarate (DMF), whose structure are also reported in **Table 1**. CURC has been extensively studied in different pathological contexts and, while to date there are no confirmed applications in humans due to the failure of clinical trials, its antioxidant properties are well-known and confirmed by a plethora of publications (Darvesh et al., 2012; Shen et al., 2013; Vera-Ramirez et al., 2013; Nabavi et al., 2015b; Serafini et al., 2017; Catanzaro et al., 2018). DMF has been approved by the Food and Drug Administration (FDA) for the treatment of relapsing-remitting multiple sclerosis and its anti-inflammatory and antioxidant properties are widely reported in literature (for an extensive review see Suneetha and Raja Rajeswari, 2016; Saidu et al., 2019).

**Table 1:** Design strategy of curcuma- and garlic-derived compounds.

Combined compounds	Derived hybrid compounds	
	Structure	Functional groups
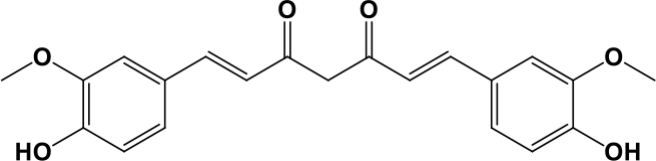 **Curcumin**	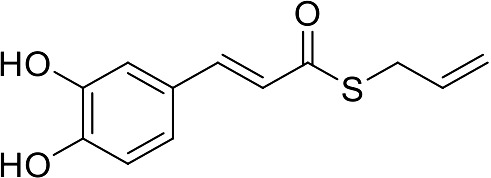 **1**	Michael acceptor Cathecol moiety Thioester Terminal double bond
	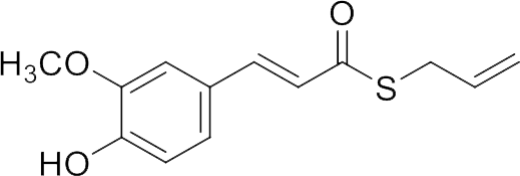 **2**	Michael acceptor Thioester Terminal double bond
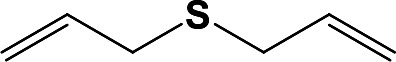 **Diallylsulfide**	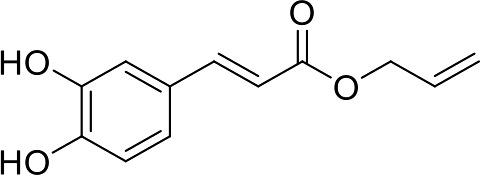 **3**	Michael acceptor Cathecol moiety Terminal double bond
	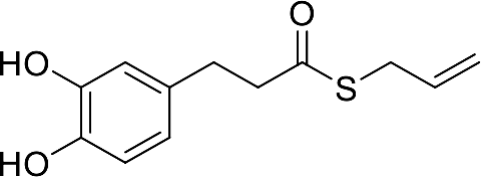 **4**	Cathecol moiety Thioester Terminal double bond
**Reference compound**	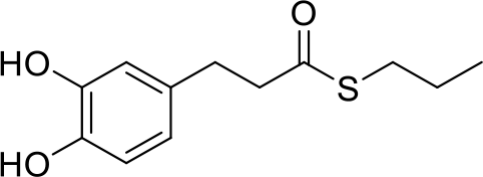 **5**	Cathecol moiety Thioester
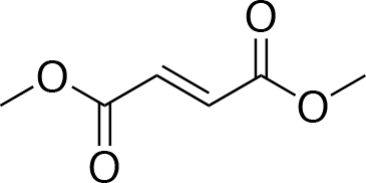 **Dimethyl fumarate**		
	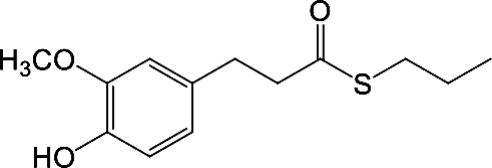 **6**	Thioester

Compound 1: S-allyl (E)-3-(3,4-dihydroxyphenyl)prop-2-enethioate;

Compound 2: S-allyl (E)-3-(4-hydroxy-3-methoxyphenyl)prop-2-enethioate;

Compound 3: allyl (E)-3-(3,4-dihydroxyphenyl)acrylate;

Compound 4: S-allyl 3-(3,4-dihydroxyphenyl)propanethioate;

Compound 5: S-propyl 3-(3,4-dihydroxyphenyl)propanethioate;

Compound 6: S-propyl 3-(4-hydroxy-3-methoxyphenyl)propanethioate

To investigate the potential interplay of compounds 1–6 with the Nrf2 cellular pathway, we first evaluated their ability to modulate the expression of the Nrf2 transcription factor and its negative regulator Kelch-like ECH-associated protein 1 (Keap1), as well as its nuclear translocation and the activation of Nrf2 downstream target genes in human neuroblastoma SH-SY5Y cells, a cell line commonly used to perform preliminary molecules screening and to dissect the underlying molecular mechanism (Narasimhan et al., 2012; Park et al., 2014; de Oliveira et al., 2019). In addition, a growing body of evidence demonstrated that several natural products, such as polyphenols, exert their protective effect through the induction of different epigenetic changes, including key modifications in microRNAs (miRNAs) expression (Howell et al., 2013; Curti et al., 2014; Boyanapalli and Kong, 2015; Liang and Xi, 2016; Curti et al., 2017; Pandima Devi et al., 2017). MiRNAs are small non-coding RNA molecules of ∼22 nucleotides in length, which are endogenously expressed and play a key role in RNA-silencing and post-transcriptional regulation of gene expression. Indeed, those noncoding RNAs modulate gene expression by suppressing translation and/or reducing the stability of their target mRNAs and consequently their target proteins. In fact, their binding to the target mRNAs, usually at the 3’-UTR, induces the recruitment of the RNA-induced silencing complex (RISC) that represses the translation of target mRNAs or enhances their cleavage (Bartel, 2004). MiRNAs can target in a combinatorial fashion a great variety of genes, which, in turn, indirectly modulate the expression of thousands of genes.

Recent studies revealed important roles of miRNAs in the control of Nrf2 activity. In addition, Nrf2 itself has been identified as a regulator of miRNAs, suggesting a loop system of mechanisms (Kurinna and Werner, 2015). In particular, miRNAs could directly target the Nrf2 mRNA and the mRNAs encoding for proteins that control the level and activity of Nrf2. As a transcription factor, Nrf2 can regulate not only the expression of protein coding parts of the genome, but also protein non-coding parts of the genome which, in turn, contains the majority of functional Nrf2-binding sites (Hirotsu et al., 2012). *In silico* analysis by Papp and colleagues predicted 85 Nrf2-miRNA interactions, with 63 miRNAs able to directly or indirectly regulate Nrf2 (Papp et al., 2012).

In line with these premises, the investigation of miRNA modulation could potentially be important in providing novel insights for a better understanding of the antioxidant activities of natural products and hybrids. Hence, we further investigated whether compounds are capable to exert epigenetic effects by modulating specific miRNAs associated with Nrf2 signaling pathway.

## Material and Methods

### Reagents

Compounds were synthesized according to previous procedures (Simoni et al., 2016; Simoni et al., 2017). Final compounds were >98% pure as determined by High Performance Liquid Chromatography (HPLC) analyses. The analyses were performed under reversed-phase conditions on a Phenomenex Jupiter C18 (150 × 4.6 mm I.D.) column, using a binary mixture of H_2_O/acetonitrile (60/40, v/v for 1, 2; 65/35, v/v for 3; 50/50, v/v for 4, 5, 6) as the mobile phase, UV detection at λ = 302 nm (for 1, 2, 3) or 254 nm (for 4, 5, 6), and a flow rate of 0.7 ml/min. Analyses were performed on a liquid chromatograph model PU-1585 UV equipped with a 20 μl loop valve (Jasco Europe, Italy). CURC (CAS number 08511) and DMF (CAS number 242926) were ≥98% and ≥97% pure respectively, and were purchased by Sigma-Aldrich (Merck KGaA, Darmstadt, Germany). All compounds were solubilized in DMSO at stock concentrations of 10 mM, frozen (−20°C) in aliquots and diluted in culture medium immediately prior to use. For each experimental setting, a stock aliquot was thawed and diluted to minimize repeated freeze and thaw damage. The final concentration of DMSO in culture medium was less than 0.1%. Cell culture media and all supplements were purchased from Sigma-Aldrich (Merck KGaA, Darmstadt, Germany). Rabbit polyclonal anti-human Nrf2 (NBP1-32822), mouse monoclonal anti-human NQO1 (NB200-209), and rabbit polyclonal anti-human HO-1 (NBP1-31341) antibodies were purchased from Novus (Biotechne, Minneapolis, USA). Mouse monoclonal anti-human Keap1 antibody (MAB3024) was purchased from R&D Systems (Biotechne, Minneapolis, USA). Mouse monoclonal anti-human β-actin (612656) and mouse anti-human lamin A/C (612162) antibodies were purchased from BD Biosciences (Franklin Lakes, NJ, USA). Finally, mouse anti-human α-tubulin (sc-5286) and mouse anti-human GSS (sc-166882) antibodies were purchased from Santa Cruz Biotechnology (Dallas, Texas, USA).

### SH-SY5Y Cell Cultures

Human neuroblastoma SH-SY5Y cells from the European Collection of Cell Cultures (ECACC No. 94030304) were cultured in a medium with equal amounts of Eagle’s minimum essential medium and Nutrient Mixture Ham’s F-12, supplemented with 10% heat-inactivated fetal bovine serum (FBS), 2 mM glutamine, 0.1 mg/ml streptomycin, 100 IU·ml penicillin and non-essential amino acids at 37°C in 5% CO_2_-containing, and 95% air atmosphere. All culture media, supplements and FBS were purchased from Sigma-Aldrich (Merck KGaA, Darmstadt, Germany).

### Cell Viability

The mitochondrial dehydrogenase activity that reduces 3-(4,5-dimethylthiazol-2-yl)-2,5-diphenyl-tetrazolium bromide (MTT, Sigma-Aldrich, Merck KGaA, Darmstadt, Germany) was used to determine cell viability using a quantitative colorimetric assay (van Meerloo et al., 2011; Kumar et al., 2018). At day 0, SH-SY5Y cells were plated in 96-well plates at a density of 2.5×10^4^ viable cells per well, respectively. After treatment, according to the experimental setting, cells were exposed to an MTT solution (1 mg/ml) in complete medium. After 4 hours of incubation with MTT, we lysed cells with sodium dodecyl sulfate (SDS) for 24 hours and cell viability was quantified by reading absorbance at 570 nm wavelength, using a Synergy HT multi-detection micro-plate reader (Bio-Tek).

### Subcellular Fractionation for Nrf2 Nuclear Translocation

The expression of Nrf2 in nuclear SH-SY5Y cell lysates was assessed by Western blot analysis. Cell monolayers were washed twice with ice-cold PBS, harvested, and subsequently homogenized 20 times using a glass-glass homogenizer in ice-cold fractionation buffer (20 mM Tris/HCl pH 7.4, 2 mM EDTA, 0.5 mM EGTA, 0.32 M sucrose, 50 mM β-mercaptoethanol). The homogenate was centrifuged at 300g for 5 minutes to obtain the nuclear fraction. An aliquot of the nuclear extract was used for protein quantification by Bradford method, whereas the remaining was boiled at 95°C for 10 minutes after dilution with 2× sample buffer (125 mM Tris-HCl pH 6.8, 4% SDS, 20% glycerol, 6% β-mercaptoethanol, 0.1% bromophenol blue). Equivalent amount of nuclear extracted proteins (30 μg) were subjected to polyacrylamide gel electrophoresis and immunoblotting as described below.

### Immunodetection of Nrf2, Keap1, NQO1, and HO-1

The expression of Nrf2, Keap1, NQO1, and HO-1 in whole cell lysates or nuclear extracts was assessed by Western blot analysis. After treatment, cell monolayers were washed twice with ice-cold PBS, lysed on the culture dish by the addition of ice-cold homogenization buffer (50 mM Tris-HCl pH 7.5, 150 mM NaCl, 5 mM EDTA, 0.5% Triton X-100, and protease inhibitor mix). Samples were sonicated and centrifuged at 13,000g for 10 seconds at 4°C. The resulting supernatants were transferred into new tubes, and protein content was determined by Bradford method. For Western blot analysis, equivalent amounts of both total and nuclear extracts (30 μg) were electrophoresed in 10% acrylamide gel, under reducing conditions, then, electroblotted into PVDF membranes (Merck KGaA, Darmstadt, Germany), blocked for 1 hour with 5% w/v bovine serum albumin (BSA) in TBS-T (0.1 M Tris-HCl, pH 7.4, 0.15 M NaCl, and 0.1% Tween 20), and incubated overnight at 4°C with primary antibodies diluted in 5% w/v BSA in TBS-T. The proteins were visualized using primary antibodies for Nrf2 (1:2,000), Keap1 (1:1,000), NQO1 (1:2,000), or HO-1 (1:2,000). Detection was carried out by incubation with secondary horseradish peroxidase-conjugated antibodies (1:5,000) diluted in 5% w/v BSA in TBS-T for 1 hour at room temperature. Membranes were subsequently washed three times with TBS-T and proteins of interest were visualized using an enhanced chemiluminescent reagent (Pierce, Rockford, IL, USA). β-Actin, α-tubulin, and lamin A/C were performed as control for gel loading.

### Real-Time PCR (RT-qPCR)

Total RNA was extracted from SH-SY5Y cells by using a RNeasy Plus Mini Kit (Qiagen, Valencia, CA, USA) following the manufacturer’s instructions. QuantiTect reversion transcription kit and QuantiTect SYBR Green PCR kit (Qiagen, Valencia, CA, USA) were used for cDNA synthesis and gene expression analysis, following the manufacturer’s speciﬁcations. Nrf2, Keap1, NQO1, HO-1, GSS, and GAPDH primers (genome wide bioinformatically validated primers sets) were provided by Qiagen (QuantiTect Primer Assays; Qiagen, Valencia, CA, USA). GAPDH was used as an endogenous reference.

### MicroRNA Analysis

After the extraction procedure, the RNA quantification was assessed using a spectrophotometric method with FLUOstar^®^ Omega (BMG LABTECH, Ortenberg, Germany) and the LVIS plate, following the operating manual instructions. RNA purity was assessed by calculating the 260/280 absorbance ratio. After quantification, a RTII Retrotrascription Kit (Qiagen) was used to promote the retrotrascription of exclusively mature miRNA following the manufacturer’s instructions. The cDNA was diluted with RNase-free water prior to start the RT-qPCR procedure. To verify the expression of miRNA targets, a miScript^®^ miRNA PCR Array (Qiagen) was used, following the manufacturer’s instructions. We performed the RT-qPCR using StepOnePlus RT-qPCR (Applied Biosystem, Foster City, California, USA). The primers were purchased from Qiagen, with specific forward primers contained in the miScript^®^ miRNA PCR Array and with reverse primers contained in the in miScript SYBR^®^ Green PCR Array. For each plate the amplification conditions were set as follows: 95°C for 15 minutes, 94°C for 15 seconds, 55°C for 30 seconds, and 70°C for 30 seconds. The last three steps were repeated for 45 cycles. SNORD61 and RNU6-6P were used as endogenous controls.

### Densitometry and Statistics

All experiments were performed at least three times. Data are expressed as mean ± SEM. The acquisition of the Western blotting images was done through a scanner and the relative densities of the bands were analyzed with ImageJ software. Statistical analyses were performed using GraphPad Software version 7.0 (La Jolla, CA, USA). Statistical differences were determined by analysis of variance (ANOVA) followed, when significant, by an appropriate *post hoc* test as indicated in figure legends. For miRNA expression, we used linear mixed models, including treatments as fixed terms and plates as random effects, which allowed for different intercepts for each run. In miRNA figures, the points indicate the mean value while the bars represent the SEM. In all reported statistical analyses, effects were designated as signiﬁcant with a *p*-value < 0.05. Statistical analyses were performed using R software version 3.4.1 (R Core Team, 2018).

## Results

### Cellular Toxicity of Curcuma- and Garlic-Derived Compounds

**Figure 1:**
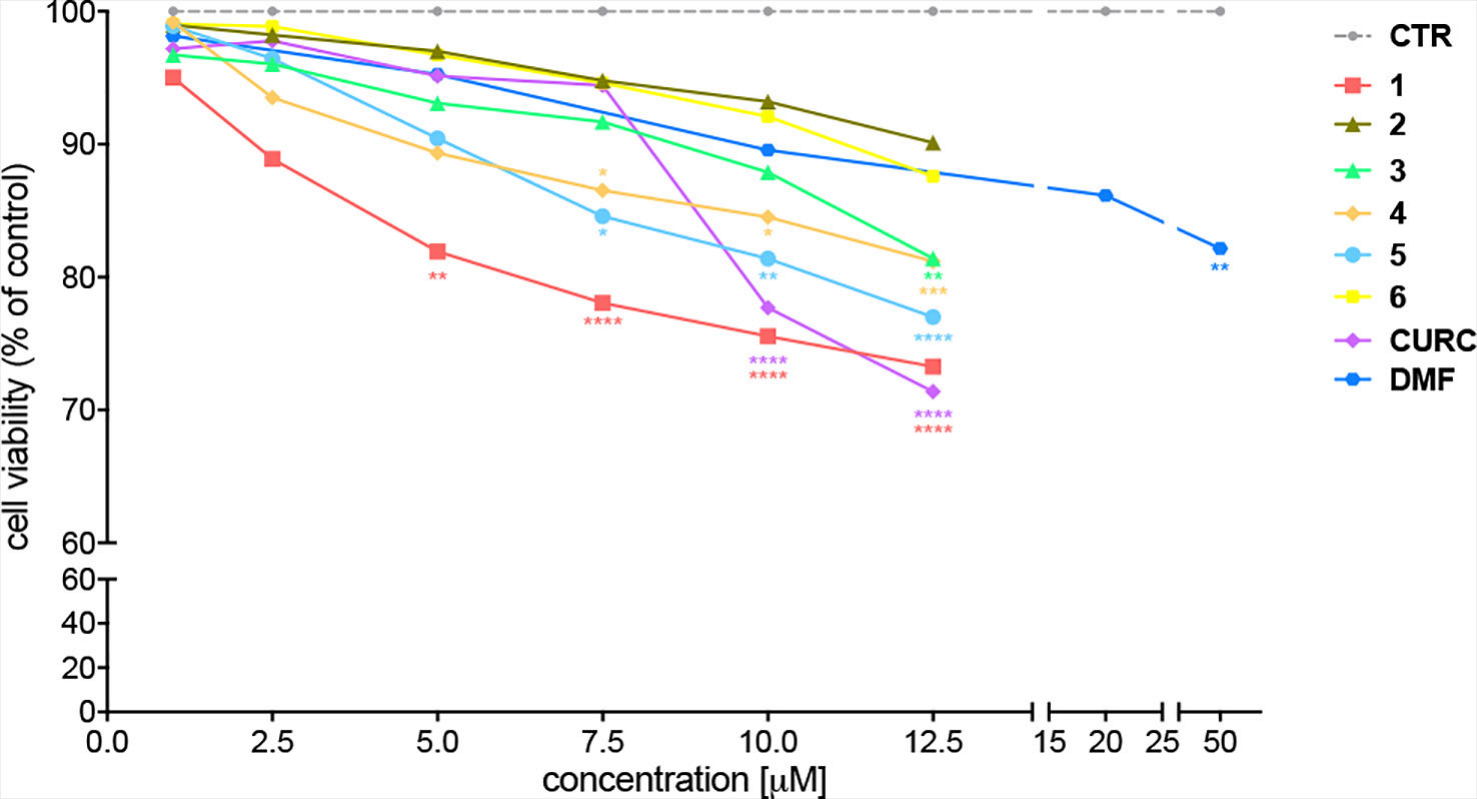
Cellular toxicity of hybrid compounds (1–6), curcumin (CURC), and dimethyl fumarate (DMF) on human neuroblastoma SH-SY5Y. Cells were treated with compounds 1–6 and CURC for 24 hours at different concentrations ranging from 1 to 12.5 μM. DMF was used in a range of concentrations starting from 0.5 μM till to 50 μM. Cell viability was assessed by MTT assay. Data are expressed as percentage of cell viability versus CTR; *p < 0.05, **p < 0.01 and ****p < 0.0001 versus CTR; Dunnett’s multiple comparison test (n ≥ 5).

The cytotoxicity of compounds 1–6 has been assessed by MTT assay in SH-SY5Y human neuroblastoma cells, in comparison with CURC and DMF. Cells were exposed to the compounds and CURC at concentrations ranging from 1 to 12.5 μM for 24 hours. The concentrations for DMF were chosen basing on literature data (Brennan et al., 2015; Campolo et al., 2018) and a range of concentrations starting from 1 μM to 50 μM has been analyzed. As shown in **Figure 1**, all the compounds were well tolerated (reduction of cell viability of about 10%) at a concentration up to 5 μM, with the exception of the prototype 1, that at 5 μM induced a slight decrease (about 20%) in cell viability, consistent with our previous data (Simoni et al., 2017).

### Modulation of Nrf2 and Its Negative Regulator Keap1

**Figure 2:**
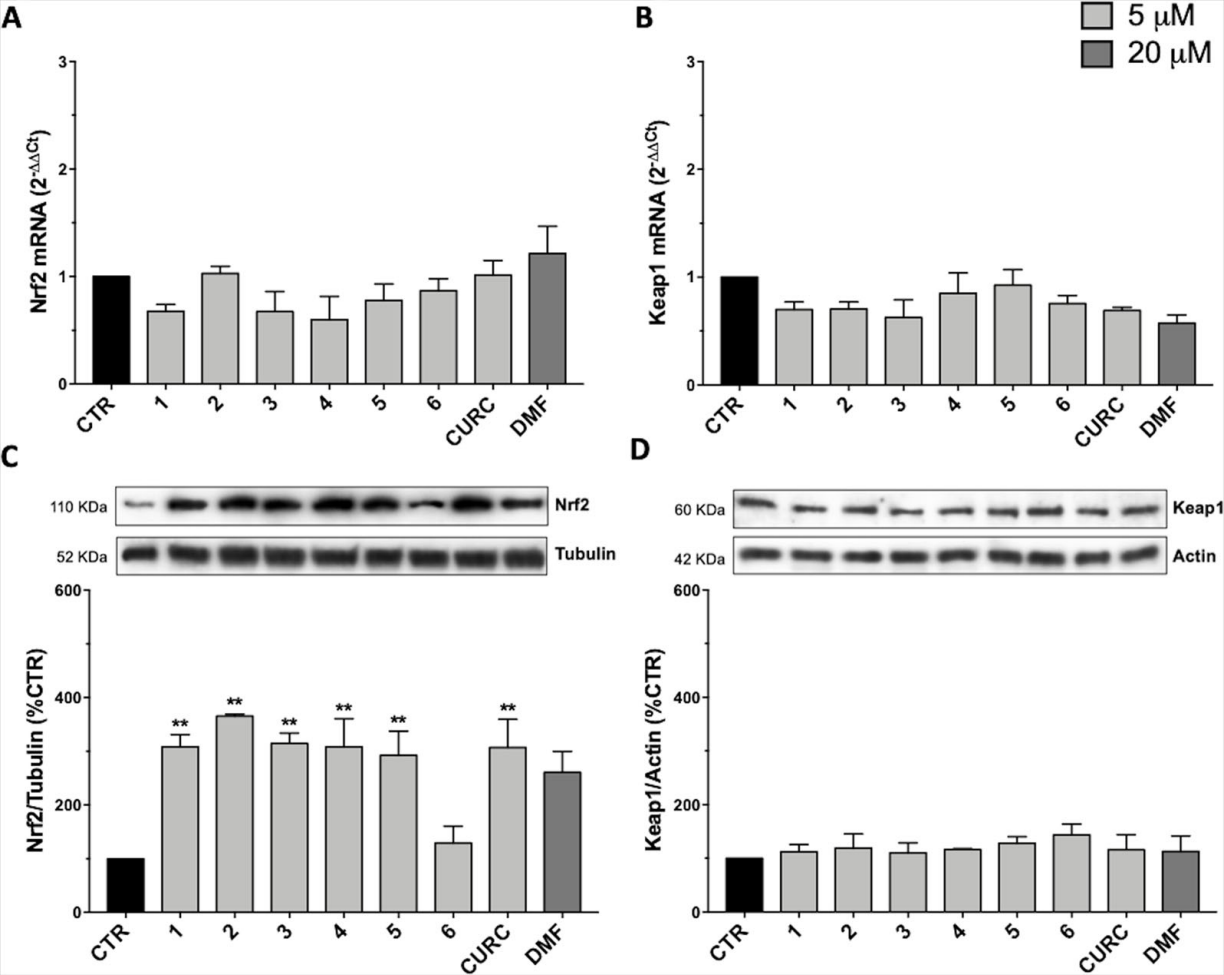
Modulation of Nrf2 and Keap1 mRNA and protein levels by compounds 1–6, curcumin (CURC), and dimethyl fumarate (DMF). **(A–B)** RNA from total cellular extracts of SH-SY5Y cells treated for 24 hours with 5 μM compounds or 20 μM DMF were analyzed for Nrf2 **(A)** and Keap1 **(B)** mRNA expression by RT-qPCR. GAPDH was used as housekeeping gene. Results are shown as mean ± SEM; no statistically significant data with Dunnett’s multiple comparison test (A, n = 3, F ratio = 1.249; B, n = 3, F ratio = 1.671). **(C–D)** Cellular extracts of SH-SY5Y cells treated for 24 hours with compounds at 5 μM or 20 μM DMF were analyzed for Nrf2 **(C)** and Keap1 **(D)** protein levels by Western blot. Anti-tubulin was used as protein loading control. Results are shown as ratio (% of CTR) ± SEM; **p < 0.01, versus CTR; Dunnett’s multiple comparison test (C, n ≥ 5, F ratio = 3.981; D, n = 3, F ratio = 0.4049).

To understand the molecular mechanisms underlying the antioxidant activity of compounds 1–6, we decided to investigate the Nrf2 pathway, which plays a key role in orchestrating cellular antioxidant defenses and in maintaining cellular redox homeostasis. To analyze the modulation of the Nrf2-mediated detoxification pathway, we performed RT-qPCR and Western immunoblotting in SH-SY5Y human neuroblastoma cells exposed to compounds 1–6 and CURC at the concentration of 5 μM or to DMF at the concentration of 20 μM for 24 hours (**Figure 2**). All compounds tested did not affect the mRNA levels of Nrf2 (**Figure 2A**) and Keap1 (**Figure 2B**), neither Keap1 protein amount (**Figure 2D**). In contrast, a strong increase in Nrf2 protein expression (**Figure 2C**) is induced by all compounds, with the exception of compound 6. DMF treatment did not produce statistically significant results in our experimental setting, although an increase trend could be assumed. Altogether, these results show that all compounds tested, with the exception of compound 6, modulate Nrf2 protein levels, but do not act at the transcriptional level.

### Nuclear Translocation of the Nrf2 Transcription Factor

Since Nrf2 nuclear translocation is an essential step for the complete activation of its pathway, we further examined the ability of the hybrids to induce the nuclear localization of Nrf2 in SH-SY5Y, by comparing their effects with CURC and DMF.

Data from literature suggest that a pro-electrophilic moiety (catechol) and/or an electrophilic moiety (the Michael acceptor α,β-unsaturated carbonyl group) are important structural functions for Nrf2 induction (Tanigawa et al., 2007; Satoh et al., 2013). The tested compounds were selected to delineate the structural requirements responsible for the activation of the transcription factor and its downstream signaling pathway. The six hybrids investigated in this study differ from each other by the presence or absence of the mentioned key functional groups (**Table 1**). Indeed, the compounds 1 and 3 provide the catechol moiety as well as the Michael acceptor group. The compounds 4 and 5 lack the Michael acceptor function but have the catechol moiety, whereas 2 shows only the Michael acceptor. The compound 6 was chosen as negative control, lacking for both Michael acceptor and catechol function. Moreover, the effects of CURC and DMF as positive controls have also been investigated.

**Figure 3:**
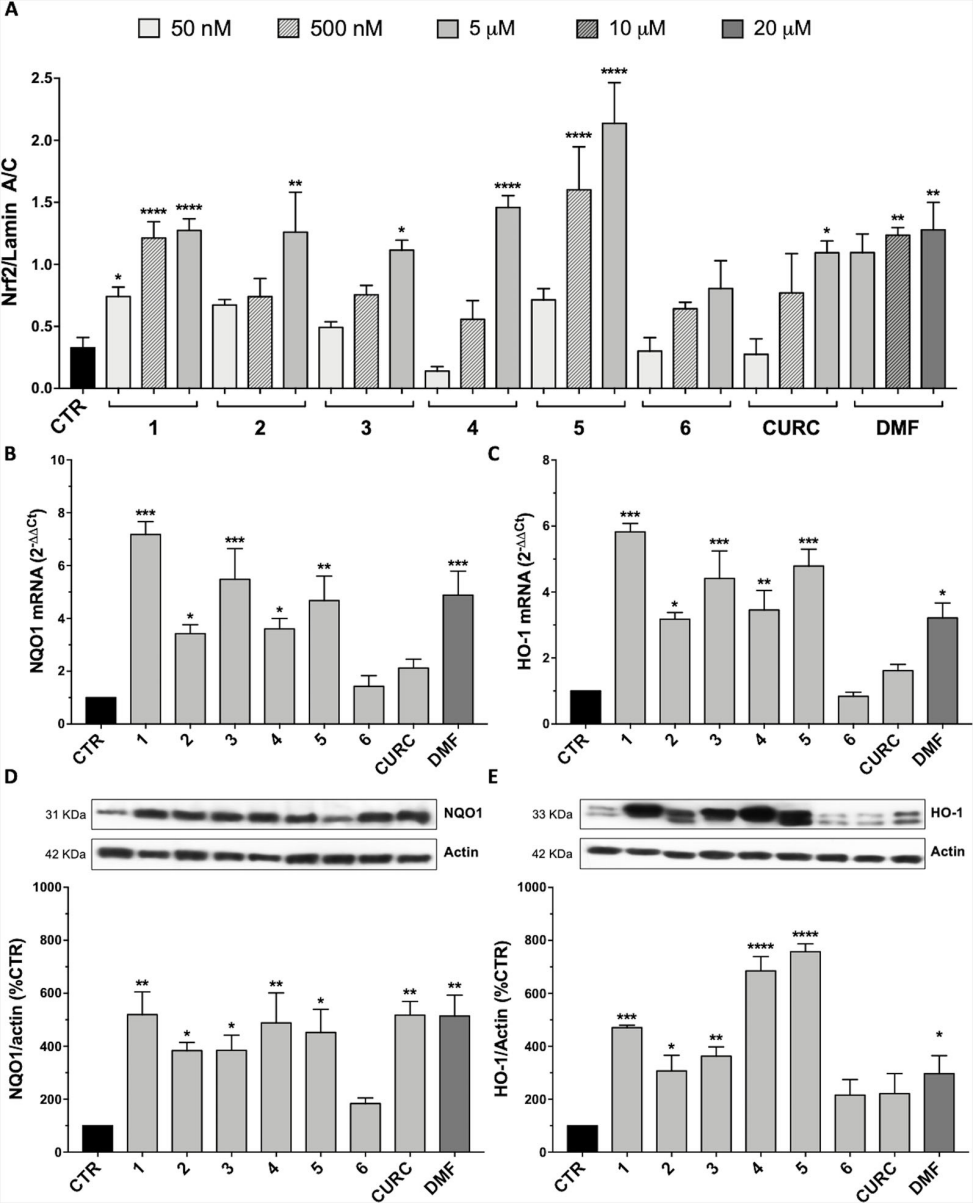
Nrf2-pathway activation by hybrids: nuclear translocation and targets induction. **(A)** Nuclear cellular extracts of SH-SY5Y cells were treated for 3 hours with compounds at 5 µM, 500 nM, and 50 nM or with 20, 10, and 5 µM dimethyl fumarate (DMF). Nrf2 protein content in the nucleus was determined by Western blot. Anti-lamin A/C was used as a protein loading control. Results are shown as ratio Nrf2/lamin A/C ± SEM; *p < 0.05, **p < 0.01 and ****p < 0.0001 versus CTR; Dunnett’s multiple comparison test (F ratio = 6.797, n≥3). **(B–C)** RNA from total cellular extracts of SH-SY5Y cells, treated for 24 hours with 5 μM compounds or 20 μM DMF, were analyzed for NQO1 **(B)** and HO-1 **(C)** mRNA expression by RT-qPCR. GAPDH was used as housekeeping gene. Results are shown as mean ± SEM; *p < 0.05, **p < 0.01, and ***p < 0.001 versus CTR; Dunnett’s multiple comparison test (B, n≥3, F ratio = 10.44; C, n≥3, F ratio = 13.95). **(D–E)** Cellular extracts of SH-SY5Y cells treated for 24 hours with compounds at 5 μM or 20 μM DMF were analyzed for NQO1 **(D)** and HO-1 **(E)** protein levels by Western blot. Anti-actin was used as protein loading control. Results are shown as ratio (% of CTR) ± SEM; *p < 0.05, **p < 0.01, ***p < 0.001, and ****p < 0.0001 versus CTR; Dunnett’s multiple comparison test (D, n ≥ 3, F ratio = 5.144; E, n≥3, F ratio = 17.26).

SH-SY5Y cells were treated with the compounds at different concentrations: 5 μM, 500 nM, and 50 nM of 1–6 and CURC or 20 μM, 10 μM, and 5 μM of DMF. As indicated in **Figure 3A**, all tested hybrids, except 6, lacking for both electrophilic features, are capable to significantly induce Nrf2 nuclear translocation at their highest concentration. This result indicates that Nrf2 nuclear translocation may rely on the presence of both the α,β-unsaturated carbonyl function and the catechol group, either alone or in combination, thus suggesting that nucleophilic addition of Keap1 cysteine residues to (pro)-electrophilic portions of the molecule might activate the Nrf2 pathway. Moreover, 1 and 5 significantly induce Nrf2 nuclear localization at the intermediate concentration of 500 nM, whereas 1 also at a concentration of 50 nM. None of the molecules, with the exception of 1, were found to act on the Nrf2 pathway at the lowest concentrations investigated (i.e., 50 nM).

### Activation of the Nrf2 Target Genes

To demonstrate the complete activation of Nrf2 pathway by the synthesized hybrids, the expression of two Nrf2 target genes has also been evaluated. Indeed, once in the nucleus, Nrf2 binds to the ARE sequences in the promoter region of its target genes, inducing the expression of phase II cyto-protective genes related to cellular stress response, such as those codifying for NQO1 and HO-1. The mRNA expression and protein levels of these two genes were evaluated by RT-qPCR and Western blot in SH-SY5Y, treated with compounds 1–6 and CURC at the concentration of 5 μM and with 20 μM DMF for 24 hours. As shown in **Figure 3**, all compounds, with the exception of 6 and CURC, induced an increase in NQO1 mRNA levels (**Figure 3B**), followed by an increase in NQO1 protein with the exception of 6 (**Figure 3D**). In a similar way, the mRNA (**Figure 3C**) and protein (**Figure 3E**) levels of HO-1 are positively modulated by all hybrids except 6, and CURC. The increase in transcription and translation of two Nrf2 target genes demonstrates the complete activation of the Nrf2 pathway.

**Figure 4:**
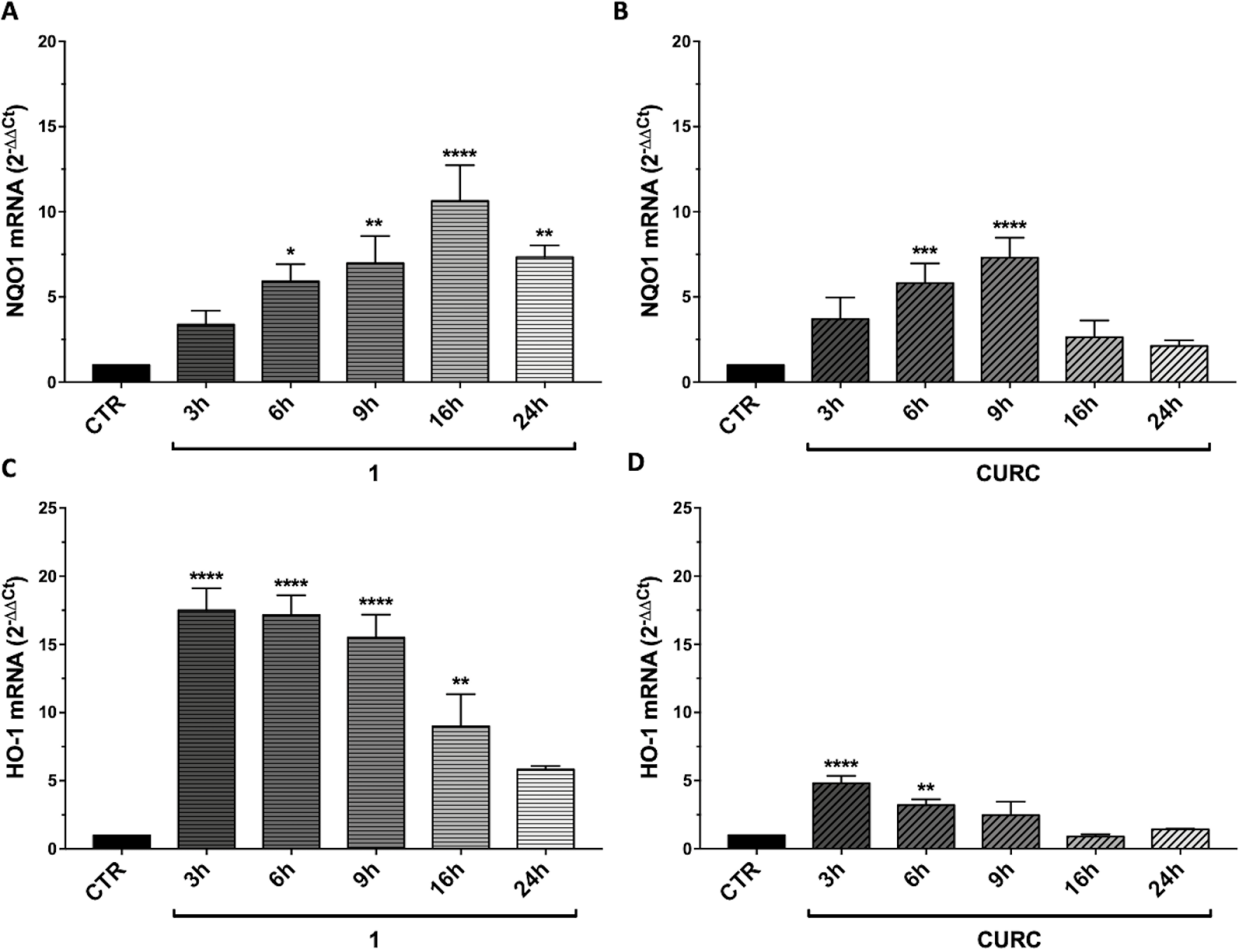
Time-dependent modulation of Nrf2 targets by compound 1 and curcumin. RNA from total cellular extracts of SH-SY5Y cells, treated for 3, 6, 9, 16, and 24 hours with 5 μM compounds 1 and curcumin (CURC), were analyzed for NQO1 **(A-B)** or HO-1 **(C–D)** relative mRNA expression by RT-qPCR. GAPDH was used as housekeeping gene. Results are shown as mean ± SEM; *p < 0.05, **p < 0.01, ***p < 0.001, and ****p < 0.0001 versus CTR; Dunnett’s multiple comparison test (A, n≥3, F ratio = 9.346; B, n≥3, F ratio = 10.44; C, n≥3, F ratio = 18.02; D, n≥3, F ratio = 13.87).

To explain the discrepancy between the obtained data showing the loss of efficacy of CURC on Nrf2 target gene activation, we further evaluated whether such result may rely on the timing of the treatment. Thus, we performed a time course using CURC and compound 1, as an example of the most active hybrid compound. SH-SY5Y cells were treated with compound 1 or CURC at the concentration of 5 μM for 3, 6, 9, 16, and 24 hours. NQO1 (**Figures 4A, B**) and HO-1 (**Figures 4C, D**) mRNAs levels were differently regulated in time, with NQO1 slowly increasing and HO-1 being boosted for 3 hours and, then, decreasing with time. Treatment with compound 1 induced a significant increase in relative NQO1 mRNA levels from 6 hours to 16 hours (**Figure 4A**), whereas CURC treatment induced an increase at 6 hours, which reached a peak at 9 hours and then lost statistical significance by 16 hours (**Figure 4B**). Treatment with compound 1 induced a strong increase in HO-1 mRNA levels, already statistically significant at 3 hours, then decreasing with time (**Figure 4C**). Here, the effect of curcumin was similar to that induced by hybrid 1, though the increase in the HO-1 mRNA levels was smaller (**Figure 4C**). Taken together, these data demonstrate that CURC induces a significant increase in NQO1 and HO-1 mRNA and protein levels at different times of treatment compared to compound 1. These results suggest that compounds may affect the Nrf2 pathway though different temporal kinetics.

### Modulation of miRNAs Related to the Nrf2 Signaling Pathway

To deepen the understanding of the mechanism through which the selected hybrids exert their antioxidant activities, in comparison to CURC, we determined the expression levels of different miRNAs in SH-SY5Y cell cultures. MiRNAs were chosen on the basis of their predicted targets with the aid of miRTarBase (http://miRTarBase.mbc.nctu.edu.tw/) an open access database which provides information about experimentally validated miRNA-target interactions (Chou et al., 2018). One single miRNA could have multiple targets, thus we focused our attention on miRNAs, which could modulate the mRNA, and consequently the protein amount, of genes involved in the Nrf2 signaling pathway, such as those codifying for HO-1 (hsa-miR-196a-5p), GSS (hsa-miR-125b-5p), and SOD2 (hsa-miR-222-3p, hsa-miR-17-3p).

SH-SY5Y human neuroblastoma cells were treated with compounds 1–6 and CURC at different concentrations (5 μM, 500 nM, and 50 nM) for 24 hours. Total RNA was extracted from treated and control cell cultures, as according to the *Material and Methods* section, and RT-qPCR assays were performed.

**Figure 5:**
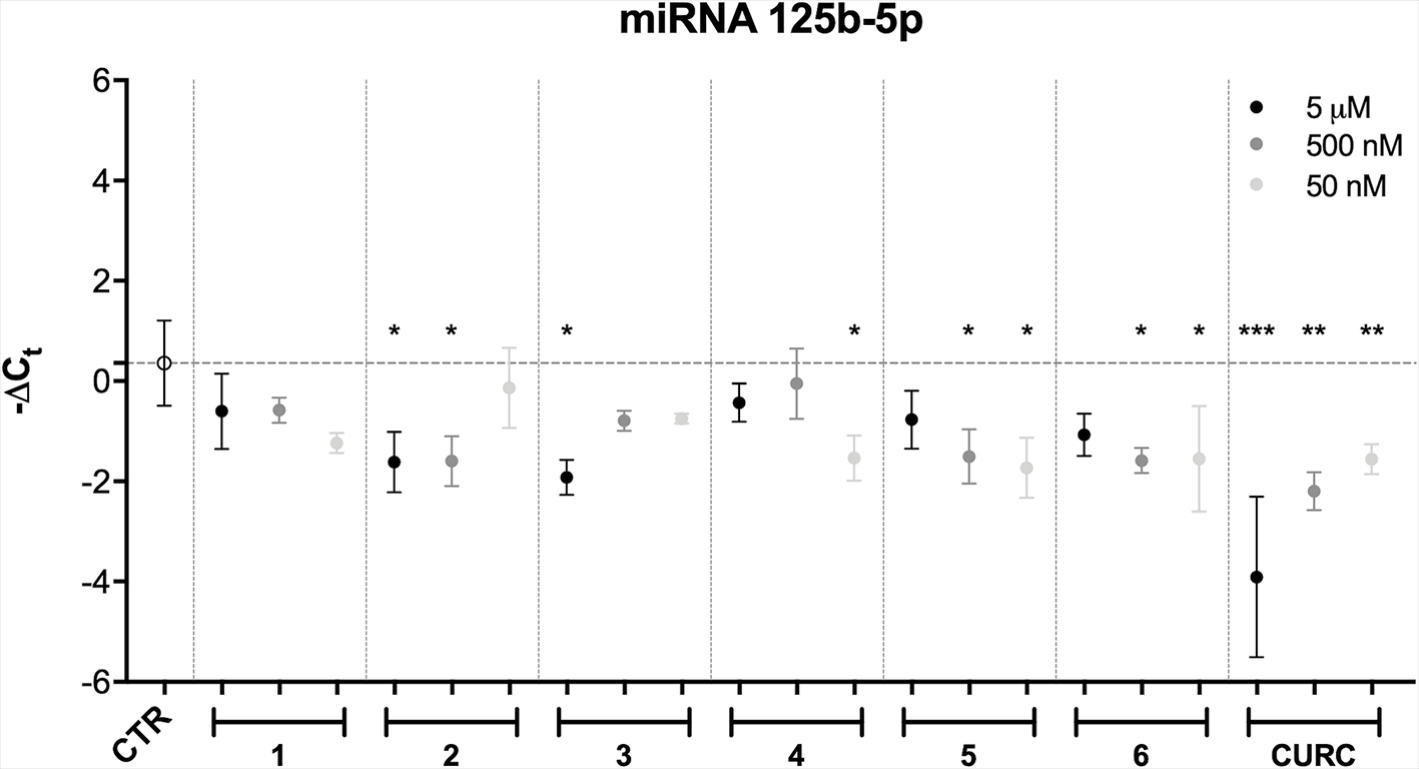
miRNA modulation by hybrids and curcumin. Expression levels (-Delta CT) of hsa-miR-125b-5p in SH-SY5Y cells treated with different newly-synthesized compounds at different concentrations (50 and 500 nM). Results are shown as mean ± SEM; *p < 0.05, **p < 0.01, and ***p < 0.001 versus CTR; Dunnett’s multiple comparison test (n = 3, F ratio = 4.584).

Among all the miRNAs analyzed, only hsa-miR-125b-5p results to be modulated with a statistically significant *p*-value. Data from literature indicate that hsa-miR-125b-5p is involved in oxidative stress, since it has mRNA coding for GSS as target (Chou et al., 2016). As far as hsa-miR-125b-5p is concerned, the results obtained following statistical analysis suggest that the expression level of this miRNA is downregulated after treatment with CURC at all concentrations (**Figure 5**). In addition, significant differences in miRNA expression levels were registered between the control and the following treatments: 5 μM and 500 nM of 2, 5 μM of 3, 50 nM of 4, 50 nM and 500 nM of 5, and 50 nM and 500 nM of 6. The decrease in miR-125b-5p after treatment with compounds 2–6 and CURC at different concentrations confirms that they have the capacity to modulate miRNAs involved in protection against oxidative stress. Nevertheless, the compounds tested did not significantly modulate the expression of mRNA coding for GSS, even if CURC at all concentrations shows an increased trend in line with the reduction of miR-125b-5p (**Table 2**). These data suggest that the process of GSS synthesis is regulated by other molecular mechanisms and the modulation of this mRNA is not strictly under the control of miR-125b-5p.

**Table 2:** GSS mRNA levels modulation. RNA from total cellular extracts of SH-SY5Y cells treated for 24 hours at different concentrations (50, 500 nM, and 5 µM) of compounds were analyzed for GSS mRNA expression by RT-qPCR. GAPDH was used as housekeeping gene. Results are shown as mean ± SEM.

GSS mRNA (2^−ΔΔCt^)			
Compound	Concentration		
	5 µM	500 nM	50 nM
**1**	0.83 ± 0.25	1.16 ± 0.12	0.61 ± 0.10
**2**	1.07 ± 0.14	1.04 ± 0.35	1.14 ± 0.05
**3**	1.17 ± 0.03	0.96 ± 0.02	1.08 ± 0.24
**4**	1.25 ± 0.22	1.31 ± 0.25	1.10 ± 0.10
**5**	1.42 ± 0.29	0.97 ± 0.25	1.90 ± 0.44
**6**	1.18 ± 0.21	1.22 ± 0.42	1.19 ± 0.23
**CURC**	1.81 ± 0.25	1.93 ± 0.45	2.24 ± 0.61

## Discussion

Nrf2 is a redox-sensitive transcription factor that has been described to play a critical role in adaptation to cellular stress and affords cellular defense by initiating transcription of antioxidant phase II and detoxification genes (Tebay et al., 2015). The hybrids here tested have been demonstrated to modulate in an *in vitro* model the activation of Nrf2-pathway and the ARE-controlled expression of its target genes codifying cyto-protective enzymes (i.e., NQO1 and HO-1).

The mechanism at the basis of the effects exerted by the compounds has been shown not to be related to the modulation in the transcription levels of Nrf2 and Keap1 (**Figures 2A, B**), as well as in the protein levels of Keap1 (**Figure 2D**), thus suggesting that in our experimental setting the increase in Nrf2 (**Figure 2C**) protein expression is not due to a decreased transcription or translation of the negative regulator Keap1. We hypothesize that compounds may directly interact with Keap1, preventing its binding to Nrf2 and, consequently, the ubiquitination process, by possibly modifying the sulfhydryl groups of cysteine residues on Keap1 and inhibiting Keap1-Nrf2 protein-protein interaction.

Subsequently, free Nrf2 in the cytoplasm could escape proteasome-targeted degradation and migrate into the nucleus to carry out its activities as a transcription factor. A proof of the hypothesis of an electrophile-based modulation of the Nrf2-pathway [consistently with what reviewed by (Basagni et al., 2019)] is the lack of efficacy in activating Nrf2 observed for compound 6, which, lacking a (pro)electrophile feature is not able to engage covalent bond with cysteine residues of Keap-1 (**Figure 3**). Combining virtual screening/molecular docking with focused exploration of structure-activity relationships (SAR) of our compounds could significantly contribute to investigate the mode of action of the hybrid compounds at a molecular level, opening prospects for further investigation. Beyond the activation of the Nrf2 pathway in a Keap1-dependent manner, data from literature further indicate that polyphenols, such as CURC, and DMF are capable to activate Nrf2 by other pathways or alternative mechanisms, including glutathione (GSH) depletion (Schmidt et al., 2007; Satoh et al., 2013; Brennan et al., 2015). GSH is known to play an important role in cellular defense against various stressors and its depletion has been also suggested to be protective against inflammation and neurodegeneration (Ewing and Maines, 1993; Aschner, 2000). Electrophiles such as curcumin and DMF have been found to induce severe side effects, due to their non-specific interaction with cysteine thiols of GSH, consequently reducing GSH levels (Satoh et al., 2013; Brennan et al., 2015). In our hand, we found that, unlike CURC, the curcuma- and garlic-inspired compounds seem not to affect the expression of GSS (**Table 2**), thus suggesting a lack of modulation in glutathione levels. This hypothesis is also supported by the results that only CURC at all the concentrations tested induces epigenetic changes through modifications in miR-125b-5p expression (**Figure 5**), in turn modulating the expression levels of mRNA coding for glutathione synthetase. Taking into account that electrophiles have complex time- and dose-dependent relationships with cellular GSH (Jobbagy et al., 2019), whether this different effect on the regulation of glutathione levels is specific only for CURC and not for our hybrids requires further investigation.

In conclusion, we have characterized, by using *in vitro* techniques, a pathway by our hybrids, which emerge as promising pharmacological tools. However, we are conscious that to translate these positive outcomes in a potential therapeutic benefit, the obtained results require to be validated in *in vivo* models. Indeed, also curcumin, whose antioxidant properties are well recognized by a plethora of publications (Darvesh et al., 2012; Shen et al., 2013; Vera-Ramirez et al., 2013; Nabavi et al., 2015b; Serafini et al., 2017; Catanzaro et al., 2018), to date does not show confirmed applications in humans due to the failure of clinical trials. Some considerations can be made on this point. A direct antioxidant effect *in vivo* may be limited by several factors, such as bioavailability, metabolic reactions, and modification of intracellular concentrations (Crespo et al., 2015). Furthermore, recent data highlight attention when referring to the use of antioxidants for supplement practice. Not only positive effects, but also negative outcomes have been observed when analyzing large numbers of studies (Visioli, 2015). As an example, the use of antioxidant mixtures (a combination of vitamins A, C, E, beta-carotene, selenium, and zinc) in the cardiovascular disease prevention has been found in several studies not to show benefits, but to result in an increase in all-cause mortality (Jenkins et al., 2018). Hence, a careful evaluation also concerning the lifestyle or other dietary factors adopted by supplement users requires multiple assessments over time.

Based on these considerations, we believe that the results here exposed evaluating the activity of hybrids 1–6 in *in vitro* studies, are promising. However, whether these profiles might result in better translational outcomes require further *in vivo* investigations to verify bioavailability issues and to test their potential in pathological models characterized by deficit in the redox system.

## Data Availability Statement

The datasets generated for this study are available on request to the corresponding author.

## Author Contributions

Conceived and designed the experiments: CL, MRo, MRa, MS, and MDag. Performed the experiments: MS, MC, FF, RC, ES, MDac, and IR. Analyzed the data: MS, MC, and FF. Critical discussion: CL, MRo, MRa, and SG.

## Funding

Research has been supported by the University of Pavia (grants from FAR, Fondo Ateneo Ricerca, to CL, MRa and MDag, from FR&G 2018, Fondo Ricerca & Giovani, to CL, MRa and SG) and the University of Bologna (grants from the RFO to MRo).

## Conflict of Interest

The authors declare that the research was conducted in the absence of any commercial or financial relationships that could be construed as a potential conflict of interest.

The reviewer RM declared a shared affiliation, with no collaboration, with one of the authors, MS, to the handling editor at time of review.
